# The Structure of Autism Spectrum Disorder Symptoms in the General Population at 18 Months

**DOI:** 10.1007/s10803-012-1546-4

**Published:** 2012-05-05

**Authors:** Karin T. Beuker, Synnve Schjølberg, Kari Kveim Lie, Rogier Donders, Martijn Lappenschaar, Sophie H. N. Swinkels, Jan K. Buitelaar

**Affiliations:** 1 Department of Psychiatry (966), Radboud University Nijmegen Medical Centre, P.O. Box 9101, 6500 HB Nijmegen, The Netherlands; 2 Karakter Child and Adolescent Psychiatry University Centre, Nijmegen, The Netherlands; 3 Division of Mental Health, Norwegian Institute of Public Health, Oslo, Norway; 4 Division of Epidemiology, Norwegian Institute of Public Health, Oslo, Norway; 5 Department of Epidemiology, Biostatistics and HTA, Radboud University Nijmegen Medical Centre, Nijmegen, The Netherlands; 6 Department of Cognitive Neuroscience, Nijmegen Centre for Evidence-based Practice, Radboud University Nijmegen Medical Centre, Nijmegen, The Netherlands; 7 Present Address: Danone Research, Wageningen, The Netherlands

**Keywords:** Autism spectrum disorders, Symptom domains, General population, Infants, MoBa, Confirmatory factor analysis (CFA), Latent class analysis (LCA)

## Abstract

It is unclear whether symptoms of autism spectrum disorder (ASD) in young children in the population fit the three-factor structure of ASD as described in the DSM-IV, and cluster together in individual subjects. This study analysed questionnaire data on ASD symptoms filled in by mothers of 11,332 18-month-old children that was collected in the context of the Norwegian Mother and Child Cohort Study conducted by the Norwegian Institute of Public Health. Confirmatory Factor Analyses showed that the three-factor model had a significantly better fit then the two- and one-factor model of ASD symptoms. Latent class analysis revealed four homogeneous groups of children (classes) with different scores for Social Interaction and Communication at one hand and Stereotypies/Rigidity at the other hand.

## Introduction

Autism spectrum disorders (ASD), which include autistic disorder, Asperger’s disorder, and pervasive developmental disorder not otherwise specified (PDD-NOS) are characterized by variations in three symptom domains, namely, deficits in social interaction, deficits in verbal and non-verbal communication, and stereotypies and rigid patterns of behavior (DSM-IV) (American Psychiatric Association 1994). It was recently reported that 9.0 per 1,000 8-year old children (95 % CI = 8.6–9.3) in the population fulfill DSM-IV criteria for ASD with a wide range in traits and severity (Centers for Disease Control and Prevention 2009). Whereas a diagnosis of autistic disorder requires the presence of symptoms in all three domains, a diagnosis of Asperger’s disorder requires deficits in social interaction and stereotypies and rigid patterns of behavior, but no clinically significant problems in early language and communication. The diagnosis of PDD-NOS, a sub-threshold or atypical manifestation of autism, is typically made on the basis of deficits in social interaction, verbal and nonverbal communication or rigid and stereotyped behaviors that do not satisfy the full set of diagnostic criteria for autism (Buitelaar and van der Gaag 1998; Buitelaar et al. 1999; American Psychiatric Association 1994). Since, there are also individuals without any ASD diagnosis, who may have specific language disorders without clinically relevant social interaction problems, and other individuals who may show obsessive–compulsive problem behavior without clinically relevant deficits in social interaction and communication, it can be questioned to what extent the three symptom domains of ASD are phenomenologically independent at different levels of symptom severity.

In a classic paper, Wing and Gould (1979) took a first step to answering this question and investigated the presence of autistic symptoms in children younger than 15 years who were included in case registers in the Camberwell area of London because of mental retardation or significant developmental problems. They found that the severity of social impairment was closely associated with abnormalities affecting comprehension and use of all forms of communication, as well as with repetitive patterns of interest (Wing and Gould 1979). Later studies using factor analysis and latent class analysis of scores for the Social Responsiveness Scale (SRS), a quantitative measure of autistic traits, showed in general population samples of children aged 7–15 years that the variation in autistic traits could be explained best by a single continuously distributed underlying factor (Constantino et al. 2000, 2003), and that distinct categories of subjects could be identified only on the basis of symptom severity (Constantino et al. 2000). This was confirmed in a clinical sample of patients with ASD and other psychiatric conditions (Constantino et al. 2004) and in siblings of children with ASD (Constantino et al. 2006). Similarly, other studies of multiplex ASD families identified subgroups on the basis of the degree of impairment (mild, moderate, or severe) across all three symptom domains of ASD rather than on the basis of distinct item endorsement profiles (Spiker et al. 2002) or significant correlations between the three symptom domains of ASD (Sung et al. 2005).

However, other multiplex ASD family and clinical sample studies reported the three symptom domains to be relatively separate from each other and in particular found deficits in social interaction to be independent of repetitive and stereotyped behaviors (Kolevzon et al. 2004; Silverman et al. 2002). Several other clinical sample studies also proposed a two factor structure of social-communicative behavior on one hand and stereotyped rigid behaviors on the other hand (Frazier et al. 2008; Gotham et al. 2007, 2008; Snow et al. 2009). The finding that joint attention in young children with ASD was associated with later social and language symptoms, but not with repetitive and stereotyped symptoms, also suggested that stereotyped and repetitive behavior may be an independent domain of ASD and have a separate developmental trajectory (Charman 2003). Further, rigid repetitive behaviors as part of the revised ADOS algorithm, make an independent contribution to diagnostic stability (Lord et al. 2006). As with the ASD multiplex studies and in clinical samples, a population-based study of 7-year-old twins found weak correlations (*r* = 0.15–0.29) between social and communicative symptoms on the one hand and non-social obsessive repetitive behaviors on the other (Ronald et al. 2005).

Studies in clinical ASD populations also suggested differences in developmental trajectory for rigid and repetitive behavior compared to other ASD symptoms. The development of social-communicative behaviors in children with ASD deviates from the typical chain of smiles and warm, joyful expressions already by 6 months, obvious interest in other people by 12 months, use of single words at 16 months, and two-word meaningful phrases by 24 months (Dietz 2007; Filipek et al. 1999). Symptoms of repetitive behaviors might be less likely to develop until the second or third year of life in children with ASD (Charman and Swettenham 2001), These symptoms are also observed with less consistency and showed more variability in young toddlers with ASD than items related to social or communication symptoms (Stone et al. 1999), and are poorly predicted from early measures of imitation or language (Charman et al. 2003; Lord and Pickles 1996). On the other hand, recent studies found signs of repetitive behaviors, as early as 12 months of age both in children with ASD and in children with typical development (Ozonoff et al. 2008; Richler et al. 2007; Thelen 1979; Watt et al. 2008), but in children with typical development these behaviors showed a general decrease after 12 months of age (Thelen 1979). A recent study by Richler (2010) also showed that low-order behaviors (repetitive sensorimotor: RSM) and high-order stereotypies (insistence on sameness: IS) have different developmental trajectories in children with ASD; RSM scores remained relatively high over time, indicating consistent severity, whereas IS scores started low and increased over time, indicating worsening (Richler et al. 2010). In sum, several studies in total populations as well as in clinical samples indicate a two factor structure of social-communicative behavior on one hand and stereotyped rigid behaviors on the other hand, and report a different developmental trajectory of these two domains.

Other studies provide support for a three factor structure. A later study in the same twins as above, conducted 1 year later, and using different measures of autistic traits showed modest phenotypic relationships between the three domains and in particular low correlations between social deficits and repetitive behaviors (Ronald et al. 2006a, b). The fractionation of the three DSM-IV ASD domains is also found in studies with clinical samples (Georgiades et al. 2007; Lecavalier et al. 2006, 2009). The discrepant findings between the above-cited studies might be due to differences in the samples (clinical versus normal population), type of instruments used to measure the ASD phenotype and procedural variations in statistical analyses (Lecavalier et al. 2009).

To date, most studies of the structure of ASD symptoms have involved older children and adults, primarily from clinical samples or high-risk populations. It is important to examine the potential fractionation of ASD domains in the general population, in addition to diagnosed populations, for two reasons. First because the clinical diagnosis of autism itself requires impairments in each of the three key areas, and this would beg the question. Second because clinical samples particularly at young age may be biased by missing cases that have not been identified due to lack of clinical concerns. Studying the distribution of ASD symptoms in the general population is justified further because ASD symptoms have been shown being on a continuum from the normal population out to individuals on the autistic spectrum (Constantino et al. 2000, 2003). Furthermore, it is not known how the three symptom domains of ASD are interrelated and cluster together in very young children in the general population.

The present study was undertaken to examine (1) whether symptoms of Autism Spectrum Disorder (ASD) in young children in the population fit the three-factor structure of ASD as described in DSM-IV, and (2) cluster together in individual subjects.

## Method

### Design

This study is based on the Norwegian Mother and Child Cohort Study (MoBa) conducted by the Norwegian Institute of Public Health (Magnus et al. 2006). In brief, MoBa is a prospective population-based pregnancy cohort study. Participants were recruited from all over Norway from 1999 to 2008, and 38.5 % of invited women consented to participate. The cohort now includes 108,000 children, 90,700 mothers and 71,500 fathers. Blood samples were obtained from both parents during pregnancy and from mothers and children (umbilical cord) at birth. Follow-up is conducted by questionnaires at regular intervals and by linkage to national health registries. Several sub-studies are conducting additional collections of data and biological materials. The current study is based on version II of the quality-assured data files released for research related to autism. Informed consent was obtained from each MoBa participant upon recruitment. The study was approved by The Regional Committee for Medical Research Ethics in the South-Eastern Netherlands.

### Participants

The study population was a subsample of the children from the MoBa study. The two inclusion criteria were: (a) the child was 18 months old during the interval 25 July 2003 to 29 March 2005 and (b) the mother had completed a questionnaire when the child was 18 months old. A total of 16,919 children met the first criterion and 13,015 children met both criteria (response rate for completion of the 18-month questionnaire was 76.6 %). Of these, individual questionnaire item data were missing for 1,683 cases, leaving 11,332 cases for analysis (mean age 18.55 ± 0.55 months; 5,776 boys and 5,522 girls, sex not reported 34 children). A full data set is required for the chosen analysis technique. Because a missing item could be due to multiple causes these 1,638 subjects did not differ from the subjects included in the analyses. In the total sample, 91.8 % of the children had parents who where both native Norwegian speakers and 87.7 % had grandparents who had Norwegian as mother tongue. The median gross income of the sample (including child support, unemployment benefits and other allowances) was NOK 200,000–299,000 ($29,000–43,000) for mothers and NOK 30,000–39,900 ($43,000–57,000) for fathers, which was higher than that of the Norwegian population overall (Median income in Norway 2003 was NOK 186,500 ($27,000) for women and NOK 285,600 ($41,000) for men (Kristiansen and Sandnes 2006).

### Procedure

This study is based on information from the questionnaire on maternal and child health, completed by the mother when the child was 18 months. A reminder was sent to non-responders after 3 weeks. All forms were scanned and the data was quality controlled and de-identified before entered in the research database (Magnus et al. 2006).

### Variables

Forty-four items that reflected ASD symptoms were selected ad hoc from the complete 18-months questionnaire used in the MoBa study. These items were originally derived from the Early Screening of Autistic Traits (ESAT) (Swinkels et al. 2006), the Modified Checklist for Autism in Toddlers (M-CHAT) (Robins et al. 2001), Ages and Stages Questionnaires (ASQ) (Squires et al. 1997), the Emotionality, Activity, Shyness, Sociability Scale (EAS scale) (Buss and Plomin 1984; Mathiesen and Tambs 1999), the Social Communication Questionnaire (SCQ; Previously called ASQ: Autism Screening questionnaire) (Berument et al. 1999), and the NonVerbal Communication Checklist (NVCC) (Schjolberg, submitted). The questions were grouped according to the three symptom domains of ASD, based on the DSM-IV and descriptions in publications of the instruments. Information about child referral to educational services, child habilitation units, and child psychiatry services until age 18 months was also obtained from the questionnaire, as was information about current and past parental worries and health problems of the child.

### Statistical approach

For analyses, all items were scored binomial. This means that answers categories were merged and recoded. The original answers were divided across two, three or five answer categories. The items with three answer categories (Yes—Sometimes—Not Yet), were recoded so that ‘Sometimes’ and ‘Not yet’ were merged. In case of five answer categories (Very typical—Quite typical—Both—Not very typical—Not typical) the two most abnormal answer categories were merged (depending on the direction of the question).

Normality of the distribution was calculated for the three ASD domains. The factor structure of ASD symptoms was examined by confirmatory factor analyses (CFA) using the computer program Mplus version 4.1 (Muthén and Muthen 2006). Three competing models were evaluated. The first model assumed that all items loaded on a single common factor. The second model made a distinction between a factor of Social Interaction/Communication symptoms and a factor of Stereotypies and Rigidity. The third model included three separate factors of Social Interaction, Communication, and Stereotypies and Rigidity. To evaluate the factor models, multiple-fit criteria were used. The Tucker–Lewis Index (TLI) and the Comparative Fit Index (CFI) reflected the improvement in fit compared to a baseline model (Bentler 1990; Marsh et al. 1988; Muthén and Muthen 2006). The TLI and CFI usually range from 0 to 1 and apply a penalty function for estimating more parameters. Larger values imply a better fit, so the model with the TLI and CFI closest to 1 was selected. For models with an acceptable fit, both the CFI and the TLI are supposed to be higher than 0.90, with the TLI value being preferably higher than 0.95. The Root Mean Square Error of Approximation (RMSEA), also an index of fit, should be <0.025 to indicate an excellent model fit. We interpreted the item scores as ordinal data and followed the approach which assumes that the observed ordinal variables stem from a set of underlying latent continuous variables. We used WLSMV (means and variance adjusted weighted least square) as estimator, because it compensates more effectively for the estimation bias that is due to the categorical aspects of the variables. The weighted least square parameter estimator uses a diagonal weight matrix with robust standard errors and mean- and variance-adjusted χ2 test statistic. (Wirth and Edwards 2007). Factor loadings should be interpreted as regression coefficients between the specific symptom and the latent construct.

In contrast to CFA, which groups items, latent class analysis (LCA) groups subjects into classes based on their item scores (Hagenaars and McCutcheon 2002). Subjects with comparable patterns of item scores, called a profile, form one class. The primary objective of LCA is to find the smallest number of classes of subjects with similar patterns of ASD symptoms that can explain the relationships among a set of observed variables. In the analysis, classes were added stepwise until the model fits the data well. Given a fixed number of classes, the deviation of each individual from the average profile of its most likely class is a measure of how well the model fits. The likelihood function of LCA is composed of two types of parameters: the marginal proportions, which are the percentages of subjects falling in each class (*γ* parameters), and the item response probabilities (*ρ* parameters). In this study, all items were binomial and scored in the same direction with a score 0 for a normal answer and score 1 for an abnormal answer. In this way, the estimated item response probabilities represent the percentage of subjects in each class reporting a particular symptom. The Bayesian Information Criterion (BIC) and the Lo–Mendell–Rubin (LMR) indexes were used to decide on the number of LCA classes, as proposed by Nylund et al. (2007). Given any two estimated models with a different number of classes, the model with the lower BIC value is the one to be preferred. The LMR compares the likelihood value of a solution with k classes to a solution with k−1 classes, providing us a *p* value to decide on significant improvement when adding an extra class to the model. At last, the interpretation of the different classes adds to the decision on how many classes in the LCA are to be preferred. LCA is always performed on a group of discrete variables, with no assumption of normality.

To examine the clinical relevance of the classes identified, the proportion of children in each class referred to developmental services, having health problems and having parents with worries about their child at 18 months was compared.

## Results

Table 1 shows the descriptive statistics (range, mean, SD) for the three domains of ASD symptoms (Social Interaction, Communication, and Stereotyped and Rigid Patterns of Behavior) by sex. Mean ± SD scores were higher for boys than for girls in all three domains (Boys: 0.98 ± 1.39; 0.64 ± .0.93; 0.93 ± 1.04; Girls: 0.85 ± 1.32; 0.37 ± 0.75; 0.88 ± 1.02; *p* values ≤ .01). The three ASD domains were non-normally distributed due to low frequencies across the answer categories.

**Table 1:** Descriptive statistics for the three domains of ASD symptoms: social interaction, communication, and stereotyped and rigid patterns of behavior by total and sex (N = 11,332)

	N	Minimum	Maximum	Median	Mean	SD
Social interaction (29 items)						
Total	11,332	0	19	1	0.92	1.35
Boys	5,776	0	17	1	0.98	1.38
Girls	5,522	0	19	0	0.85	1.32
Communication (7 items)						
Total	11,332	0	8	0	0.51	0.86
Boys	5,776	0	8	0	0.64	0.93
Girls	5,522	0	8	0	0.37	0.75
Stereotyped and rigid patterns of behavior (8 items)						
Total	11,332	0	6	1	0.91	1.03
Boys	5,776	0	6	1	0.93	1.04
Girls	5,522	0	6	1	0.88	1.02

Total N includes 5,776 boys, 5,522 girls and 34 children with sex not reported

### Confirmatory Factor Analysis

**Fig. 1:**
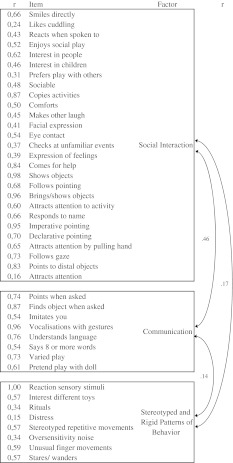
Correlations of items on the corresponding factor and correlations between factors

To test the three competing models, first the model with three factors was analyzed. The three-factor model of ASD symptom scores at age 18 months had a relatively good fit with (CFI = 0.889, TLI = 0.947, RMSEA = 0.018). There was a significant correlation between Social Interaction (factor 1) and Communication (factor 2) (*r* = 0.46), and weak but still significant correlations between Stereotyped and Rigid Patterns of Behavior (factor 3) and both Social Interaction (*r* = 0.17) and Communication (*r* = 0.14) (see Fig. 1).

The three-factor model had a slightly, but still significantly better fit than the two-factor model (CFI = 0.885, TLI = 0.946, RMSEA = 0.019) with Social Interaction and Communication combined as one factor and Stereotyped and Rigid Patterns of Behavior as the other factor (*χ*² = 67.73, *df* = 2 *p* < .0001). The correlation between the factors in the two-factor model was very low but significant (*r* = 0.15). The one-factor model had a significantly poorer fit (CFI = 0.830, TLI = 0.926, RMSEA = 0.022) than the two- (*χ*² = 271.91, *df* = 1 *p* < .0001) and three-factor (*χ*² = 349.03, *df* = 1 *p* < .0001) models and was therefore not preferred.

### Latent Class Analysis

The LCA analyses used the 44 ASD items as independent variables and identified four classes based on the BIC and LMR indexes and the interpretation of the classes. BIC values showed minor differences between the several LCA using different number of classes (Table 2). The LMR with four compared to five was significant (*p* < .01), but the interpretation of the additional class did not add much to the interpretation of the pattern of classes. The LMR with five compared to six classes was not significant (*p* > .01).

**Table 2:** Bayesian information criterion (BIC) and the Lo–Mendell–Rubin (LMR) indexes by number of classes used in the LCA

k classes	BIC	LMR	*p* (k − 1, k)
3	153,933.33	1,739.88	.000
4	153,028.81	1,188.02	.000
5	152,637.11	795.89	.005
6	152,659.94	386.20	.014

**Fig. 2:**
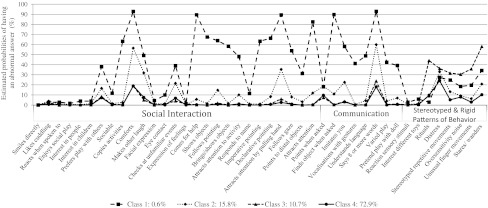
Estimated item response probabilities (%) for four-class latent class analysis model

Estimated item response probabilities for abnormal answers were calculated as the percentage of subjects in each class reporting a particular symptom (Fig. 2). High scores indicate more symptoms of difficulty in that domain, in other words high scores mean less social interaction and communication skills and more stereotyped and rigid patterns of behavior. Class 1 (0.6 % of sample) had the highest scores for both Social Interaction and Communication, but moderately high scores for Stereotyped and Rigid Patterns of Behavior. The second class (15.8 % of sample) had scores for both Social Interaction and Communication that were in-between those of class 1 and class 4, but had very low scores for Stereotyped and Rigid Patterns of Behavior. Class 3 (10.7 % of sample) had normal, i.e. baseline level, scores for Social Interaction as well as Communication, but very high scores for Stereotyped and Rigid Patterns of Behavior. The fourth and last class (72.9 % of sample) had low scores for all three symptom domains and was considered the reference group.

Some items were better than others in discriminating between classes. The estimated item response probabilities (%) by class are shown in Fig. 2 and listed in Table 3. Within the domain of social interaction, the joint attention items, and the items on copying activities and comforting the parent discriminated the best between class 1 and the other classes. All items within the domain of Communication showed a very large difference between the probability of having an abnormal answer in class 1 compared to the other classes. Within the domain of Stereotyped and Rigid Patterns of Behavior, a high proportion of children in class 3 showed an abnormal answer on the item “rituals”, compared to children in the other classes. The items “stereotyped repetitive movements”, “oversensitivity for noise”, “unusual finger movements”, and “stares/wanders” showed relatively high scores for class 3 and moderate probability scores for class 1.

**Table 3:** Estimated item response probabilities (%) for each item by class (N = 11,332)

	Item*	Class 1: 0.6 %	Class 2: 15.8 %	Class 3: 10.7 %	Class 4: 72.9 %	Sign χ²
Social interaction	Smiles directly^1^	0.0	0.0	0.0	0.0	
	Likes cuddling^1^	2.7	3.9	2.3	0.7	d,e,f
	Reacts when spoken to^1^	2.8	1.0	0.0	0.2	b,c,d,e
	Enjoys social play^1^	1.7	0.7	0.4	0.0	e,f
	Interest in people^1^	3.9	0.3	0.2	0.0	a,b,c,e
	Interest in children^2^	3.9	1.6	0.3	0.1	b,c,d,e
	Prefers play with others^5^	37.9	16.6	8.3	7.3	a,b,c,d,e
	Sociable^5^	11.8	1.6	0.7	0.2	a,b,c,e
	Copies activities^3^	63.3	2.6	0.3	0.1	a,b,c,d,e
	Comforts^6^	92.7	56.5	18.9	19.0	a,b,c,d,e
	Makes other laugh^7^	49.1	31.9	5.0	7.5	a,b,c,d,e,f
	Facial expression^1^	4.3	1.2	0.1	0.1	b,c,d,e
	Eye contact^1^	9.9	1.5	0.8	0.1	a,b,c,e,f
	Checks at unfamiliar events^2^	38.9	21.6	4.1	6.8	a,b,c,d,e,f
	Expression of feelings^1^	1.4	0.4	0.9	0.0	e,f
	Comes for help^3^	89.4	5.7	0.3	0.3	a,b,c,d,e
	Shows objects^1^	67.4	1.6	0.0	0.0	a,b,c,d,e
	Follows pointing^2^	63.8	14.8	2.0	0.7	a,b,c,d,e,f
	Brings/shows objects^2^	58.3	1.6	0.0	0.0	a,b,c,d,e
	Attracts attention to activity^2^	48.0	10.1	0.3	1.1	a,b,c,d,e,f
	Responds to name^2^	11.4	1.1	0.1	0.0	a,b,c,d,e
	Imperative pointing^3^	63.3	1.1	0.2	0.0	a,b,c,d,e,f
	Declarative pointing^2^	66.3	8.3	1.1	0.7	a,b,c,d,e
	Attracts attention by pulling hand^3^	89.3	35.5	6.1	2.6	a,b,c,d,e
	Follows gaze^4^	53.8	8.0	0.6	0.9	a,b,c,d,e
	Points to distal objects^4^	31.2	3.3	0.0	0.0	a,b,c,d,e
	Attracts attention^1^	82.4	12.0	0.4	0.2	b,d,e
Communication	Points when asked^3^	18.0	18.7	7.5	9.7	a,b,c,d,e,f
	Finds object when asked^3^	89.6	11.0	0.4	0.2	a,b,c,d,e
	Imitates you^2^	58.0	22.6	3.6	3.1	a,b,c,d,e
	Vocalizations with gestures^4^	41.1	0.7	0.1	0.0	a,b,c,d,e
	Understands language^2^	48.7	4.4	0.3	0.1	a,b,c,d,e
	Says 8 or more words^3^	92.9	60.0	23.9	18.0	a,b,c,d,e,f
	Varied play^1^	42.4	4.0	0.6	0.1	a,b,c,d,e
	Pretend play with doll^3^	39.2	7.0	1.0	0.7	a,b,c,d,e
Stereotyped and rigid patterns of behavior	Reaction sensory stimuli^1^	2.8	0.6	0.2	0.0	c,e
	Interest different toys^1^	5.9	1.5	0.7	0.3	b,c,e
	Rituals^6^	2.9	8.7	44.2	9.7	b,d,f
	Distress^3^	27.4	33.1	36.3	24.1	d,e,f
	Stereotyped repetitive movements^1^	24.7	13.2	31.7	5.2	b,c,d,e,f
	Oversensitivity noise^2^	18.4	9.3	30.5	7.9	b,c,d,f
	Unusual finger movements^2^	19.7	6.3	35.8	2.9	a,b,c,d,e,f
	Stares/wanders^2^	34.2	20.9	58.2	10.3	b,c,d,e,f

a = significant difference between class 1 and 2 (*p* < .01); b = significant difference between class 1 and 3 (*p* < .01); c = significant difference between class 1 and 4 (*p* < .01); d = significant difference between class 2 and 3 (*p* < .01); e = significant difference between class 2 and 4 (*p* < .01); f = significant difference between class 3 and 4 (*p* < .01)

* Items were derived from: ^1 ^Early Screening of Autistic Traits (ESAT; Swinkels et al. 2006); ^2 ^Modified Checklist for Autism in Toddlers (M-CHAT; Robins et al. 2001); ^3 ^Ages and Stages Questionnaires (ASQ; Squires et al. 1997); ^4 ^NonVerbal Communication Checklist (NVCC; Schjolberg, submitted); ^5 ^Emotionality, Activity, Shyness, Sociability Scale (EAS scale; Buss and Plomin, 1984; Mathiesen and Tambs 1999); ^6 ^Social Communication Questionnaire (SCQ; Previously called ASQ: Autism Screening questionnaire; Berument et al. 1999)

Class 1 consisted of 55.9 % boys and 44.1 % girls; class 2 of 62.7 % boys and 37.3 % girls; class 3 of 52.4 % boys and 47.6 % girls; and class 4 of 48.4 % boys and 51.6 % girls. The clinical relevance of the classes was further explored by looking at referral status at 18 months, health problems and parental worries (Table 4). Children in class 1 were by far most often referred to all three clinical developmental services, but in particular to Educational Services and Child Habilitation Units. More children in class 2 than in class 4 were referred to Educational Services and Child Habilitation Units. The proportion of health problems, such as delayed motor development (71.4 %) and delayed or aberrant language (60.9 %) and parental worries about the child’s physical development (45.1 %), behavior (22.5), and hearing (22.5 %) also was by far the highest in class 1.

**Table 4:** Proportion of children referred to developmental services, having health Problems, and parental worries within each class (N = 11,332)

Item	Class 1: 0.6 %	Class 2: 15.8 %	Class 3: 10.7 %	Class 4: 72.9 %	Sign χ²
Any developmental services	62.0	2.0	1.1	0.8	a,b,c,d,e
Educational services	54.3	0.8	0.6	0.1	a,b,c,e,f
Child habilitation unit	48.5	1.1	0.2	0.4	a,b,c,d,e
Child psychiatry services	3.0	0.5	0.3	0.3	a,b,c
Health problems	72.9	10.7	5.6	4.1	a,b,c,d,e,f
Hearing	11.6	3.0	1.9	1.5	a,b,c,e
Delayed motor development	71.4	6.7	2.8	2.4	a,b,c,d,e
Diverging head circumference	26.9	3.7	2.7	2.7	a,b,c,e
Delayed or aberrant language	60.9	3.2	1.3	0.4	a,b,c,d,e,f
Seizures 6–18 months	2.9	0.3	0.3	0.3	a,b,c
Worries	60.6	15.3	14.7	10.4	a,b,c,e,f
Physical development	45.1	2.8	1.7	0.7	a,b,c,e,f
Behavior	22.5	2.1	2.2	0.6	a,b,c,e,f
Difficult to handle	7.0	2.9	3.2	1.0	c,e,f
Hearing	22.5	2.1	1.2	1.1	a,b,c,e
Other	47.1	10.7	10.3	9.1	a,b,c,e

a = significant difference between class 1 and 2 (*p* < .05); b = significant difference between class 1 and 3 (*p* < .05); c = significant difference between class 1 and 4 (*p* < .05); d = significant difference between class 2 and 3 (*p* < .05); e = significant difference between class 2 and 4 (*p* < .05); f = significant difference between class 3 and 4 (*p* < .05)

## Discussion

In this large population based study (N = 11,332) among 18-month-old children the underlying structure of ASD symptoms was found generalizable to the general population at very young age. In the confirmatory factor analysis the three-factor model had a significantly better fit than the two- and one-factor model. Latent class analyses identified four classes based on the presence of different autistic symptoms, and with a distinction between the presence of social interaction and communication symptoms versus stereotypies and rigidity symptoms. These classes could further be differentiated by referral status, health problems and parental worries about the child at 18 months.

The continuous singular factor for ASD symptoms suggested by Constantino et al. (2000, 2003, 2004) was not confirmed in the CFA by the current study. The one-factor model had a significantly poorer fit to the data than the two- and three-factor models, in which Social Interaction and Communication seemed to be closely associated, and in turn were only weekly associated with Stereotyped and Rigid Patterns of Behavior. Indeed, the analyses suggested that Stereotyped and Rigid Patterns of Behavior is a distinct domain. However, methodological differences may underlie the discrepancy between our findings and those of Constantino et al. For example, Constantino et al. looked at older children, used different items and performed bottom-up based exploratory factor analysis (EFA), whereas we used a top-down CFA. EFA of the current data or CFA of the SRS data of Constantino et al. (2000, 2003, 2004) is needed to enable appropriate comparison of the findings of the two studies.

Based on fit indices, the two factor model of social/communication items and rigid repetitive behaviors was quite similar to the three-factor solution and better than the one-factor solution. The significant difference between the CFA fit indexes of the two and three factor model should be interpreted with some prudence due to the large sample size by which the significance levels were easily reached. Because the current data does not provide an unequivocal case for the two or three factor model of ASD symptoms and a two factor solution (with overlap between social and communication items) also has been reported in previous research, the similarity between the two factor model and the widely accepted three-factor DSM-IV model at least warrants discussion. The weak correlation between social (social interaction and communicative impairment) and non-social (rigid repetitive) behaviors in this study is consistent with the results of earlier population-based studies of twins at age 7 (Ronald et al. 2005) and age 8 (Ronald et al. 2006a, b). However, our finding of a relatively strong association between Social Interaction and Communication symptoms was not reported in the twin study (Ronald et al. 2006a, b), which showed modest phenotypic correlations between all three ASD symptom domains. This difference might be due to the relevance of the DSM-IV criteria to children of different ages (18 months in the present study versus 8 years in the twin study). According to the DSM-IV, gestures, non-verbal behavior and joint attention skills are part of the domain of Social Interaction; however, 18-month-old children have a very limited use of expressive language and non-verbal communication plays a greater role than it does in older children, where the distinction between Social Interaction and Communication may be more clear-cut.

Of the four classes identified by LCA on the basis of the autistic symptom profile, children classified in class 1 had high scores for all symptom domains, in particular on Social Interaction and Communication. Joint attention and language/communication items distinguished this class from the other classes. The high scores of the subjects of this class on social interaction and communication problems along with increased scores for repetitive behaviors suggest a similarity with high-risk or even clinical ASD children. This idea is supported by the finding that a high proportion of the children in this class were referred to educational services and child habilitation units. However, for now in the absence of final diagnoses, we could only confirm the high referral rate in this class, with children scoring high on autistic traits.

Class 2 (15.8 % of the sample) could represent a sub-clinical class with somewhat elevated scores on symptoms of Social Interaction and Communication and low scores on symptoms of Stereotyped and Rigid Patterns of Behavior. The proportion of children from class 2 who were referred to specialized services was only slightly higher than that of class 4, the reference group. One may hypothesize that some of these children from class 2 might later present with the broad ASD phenotype, or be diagnosed with milder forms of ASD, language disorders, or mental retardation.

Class 3 (10.7 % of the population) had a different profile of ASD symptoms by having high scores for symptoms of Stereotyped and Rigid Patterns of Behavior, but baseline scores for Social Interaction and Communication. The way Stereotyped and Rigid Patterns of Behavior seemed to be separated from the other two ASD domains was demonstrated by both the CFA on the level of items (factors) and the LCA in the clustering of individuals, and might be consistent with a study by Charman (2003), who reported that the developmental trajectory for stereotypic behavior might be different from that for social and language deficits in ASD. However, although well-described in ASD (American Psychiatric Association 1994; Lewis and Bodfish 1998; Watt et al. 2008), repetitive and stereotypic behavior is also seen among individuals with mental retardation and other disorders (Bodfish et al. 1995, 2000; Lender et al. 1998) as well as in typically developing infants and children (Foster 1998; Leekam et al. 2007; MacDonald et al. 2007; Thelen 1979; Troster 1994). While stereotyped behavior in typically developing children becomes less varied and less frequent or remains stable with increasing age (Thelen 1979), it increases with age in children with ASD (MacDonald et al. 2007), particularly high-order stereotypies like rituals and insistence on sameness (Richler et al. 2010). The overall development and stability (increase, decrease, or remain the same) of these stereotypies will show whether the children of class 3 have a typical or aberrant development. For example, the hypothesis should be tested that these children might have an increased risk of developing an obsessive and rigid temperament, as described by Garland and Weiss (1996). Separating low order behaviors from high order stereotypies could also add to the knowledge about developmental trajectories of stereotyped and rigid patterns of behavior and thereof of identifying an aberrant development. For the moment, the behavior of these children was apparently not perceived by the parents or kindergarten as warranting referral to specialized services.

There were more boys than girls in the two supposedly clinical classes (classes 1 and 2), but the male predominance was not as great as that found among ASD cases at a later age. For example, Baird et al. (2006) found a male: female ratio of 3.3:1 at 9 and 10 years for all ASD with an overall prevalence of 1.2 %.

The results of this study should be interpreted in the context of its strengths and limitations. A strength of this study is the large and homogeneous sample of very young subjects. A possible limitation may be that the results of our multivariate analyses are dependent on and limited by the various items chosen to represent the three domains. The majority of the relevant ASD items were from the Social Interaction domain. Fewer items of the Communication and Stereotyped and Rigid Patterns of Behavior domains were included, which means that some ASD symptoms in these domains might not have been fully covered, which could have biased the outcome of the CFA. However, the accuracy of the factor analysis does not only depend on the number of items in a factor, but also for example on the relation between the items and on the extent the items reflect behaviors that are easily assessed by parents. Another possible limitation is that information about symptoms were based on parent reports only. The use of more objective measures, such as test results or more formal diagnostic observations and procedures, might have led to other results. However, parents are the main and in almost all cases sole informant about very young children’s behavior problems, and there is a body of evidence indicating the merit and validity of parent information. Previous studies by Glascoe (1999, 2003) and Tervo (2005) for example, found that parental concerns relate directly to their child’s wellbeing and development. Our prior work on population screening on autism spectrum disorders found that parental judgment about whether or not to comply with professional recommendations did reflect a rather accurate estimate of the severity of autistic symptoms of their child (Dietz et al. 2007).

This population-based study showed that ASD symptoms cluster together in the three domains as defined by the current classification system DSM-IV, and can be retrieved in our latent classes with subjects with a similar profile of ASD symptoms. At least one of these classes includes subjects with high scores on social interaction and communication problems and increased score of repetitive and stereotyped behaviors rather similar to high-risk or even clinical ASD cases. This information is relevant to improve current screening instruments and screening methods. It is for example of interest to know how many children show certain behavior in the general population to establish the point at which this behavior is considered abnormal. Further, since the validity of the distinction between ASD subtypes is unclear and the current diagnostic criteria in the DSM-IV are under discussion, these results can add to more knowledge on the development of the new DSM-V. Further follow-up of this cohort is required to examine how the classes develop with age and to characterize the children included in each class in terms of measures of external validity, such as cognitive and language skills, temperament, measure of neural structure and function, impairment of psychosocial functioning, and family loading for psychiatric disorders.
